# In vitro T cell responses to PD-1 blockade are reduced by IFN-α but do not predict therapy response in melanoma patients

**DOI:** 10.1007/s00262-024-03760-z

**Published:** 2024-07-05

**Authors:** Laura M. Timmerman, Lobke C. M. Hensen, Mick J. M. van Eijs, Rik J. Verheijden, Karijn P. M. Suijkerbuijk, Linde Meyaard, Michiel van der Vlist, the UNICIT consortium, Linde Meyaard, Jürgen H. E. Kuball, Bas Oldenburg, Jeanette H. W. Leusen

**Affiliations:** 1 Center for Translational Immunology, University Medical Center Utrecht, Utrecht University, Utrecht, The Netherlands; 2 Oncode Institute, Utrecht, The Netherlands; 3 Department of Medical Oncology, University Medical Center Utrecht, Utrecht University, Utrecht, The Netherlands

**Keywords:** PD-1 blockade, Type I IFN, MLR, Melanoma

## Abstract

PD-1 blockade therapy has revolutionized melanoma treatment, but still not all patients benefit and pre-treatment identification of those patients is difficult. Increased expression of inflammatory markers such as interleukin (IL)-6 in blood of patients correlates with poor treatment response. We set out to study the effect of inflammatory cytokines on PD-1 blockade in vitro. For this, we studied the effect of IL-6 and type I interferon (IFN) in vitro on human T cells in a mixed leukocyte reaction (MLR) in the absence or presence of PD-1 blockade. While IL-6 reduced IFN-γ secretion by T cells in both the presence and absence of PD-1 blockade, IFN-α specifically reduced the IFN-γ secretion only in the presence of PD-1 blockade. IFN-α reduced T cell proliferation independent of PD-1 blockade and reduced the percentage of cells producing IFN-γ only in the presence of PD-1 blockade. Next we determined the type I IFN score in a cohort of 22 melanoma patients treated with nivolumab. In this cohort, we did not find a correlation between clinical response and type I IFN score, nor between clinical response and IFN-γ secretion in vitro in a MLR in the presence of PD-1 blockade. We conclude that IFN-α reduces the effectiveness of PD-1 blockade in vitro, but that in this cohort, type I IFN score in vivo*,* nor IFN-γ secretion in vitro in a MLR in the presence of PD-1 blockade correlated to decreased therapy responses in patients.

**Supplementary Information:**

The online version contains supplementary material available at 10.1007/s00262-024-03760-z.

## Introduction

PD-1 blockade revolutionized treatment of many cancer types by unleashing anti-tumor T cell responses that clear the tumors [1–3]. However, not all patients benefit from PD-1 blockade as treatment response varies heavily between patients, and some patients develop severe side effects known as immune-related adverse events [4–6]. Hence, prediction of PD-1 blockade treatment response would greatly benefit patient care.

PD-1 is expressed on various immune cells, including monocytes, natural killer (NK) cells, B cells and T cells [7]. PD-1 is expressed on exhausted T cells and upregulated on naïve T cells following activation. Moreover, PD-1 provides negative feedback during T cell activation [7]. PD-1 inhibits T cell receptor (TCR) signaling [7] and costimulatory signaling by CD28 [8], and ligation results in decreased cytokine production, proliferation and survival of T cells [9]. The ligands for PD-1, PD-L1 and PD-L2 are expressed by stromal cells and immune cells. Within the immune system, PD-L1 and PD-L2 are expressed by myeloid dendritic cells (DCs), and PD-L1 is expressed on plasmacytoid DCs and activated T cells [7]. In the tumor microenvironment, PD-L1 and PD-L2 expressed by tumor cells suppress tumor infiltrating T cells [10]. Hence, therapeutic blockade of PD-1 removes a brake on anti-tumor responses by T cells.

PD-1 has two intracellular signaling domains, one immunoreceptor tyrosine-based inhibitory motif (ITIM) and one immunoreceptor tyrosine-based switch motif (ITSM) [11]. PD-1 predominantly recruits the phosphatase SHP-2 to suppress T cell activation [12]. The ITSM motif is shared with signaling lymphocytic activation molecule (SLAM)-family receptors that can relay either activating or inhibiting signals, depending on the presence of SLAM-associated protein (SAP) [13]. SAP can interfere with PD-1 function by protecting the tyrosine residues of PD-1 required for signaling from deactivation by SHP-2 [14]. Intriguingly, exposure to interferon (IFN)-α, interleukin (IL)-2, IL-12 and poly-I:C upregulates SAP expression and downregulates SHP-2 expression in NK cells [15], which may suggest a link between inflammation and PD-1 function and possible PD-1 blockade treatment outcome.

In melanoma patients, high serum concentrations of C-reactive protein (CRP) and the proinflammatory cytokine IL-6 before start of PD-1 blockade therapy correlate with poor treatment outcome [16–21]. In addition, type I IFN signaling has been associated with resistance to PD-1 blockade therapy in vivo through induced expression of nitric oxide synthase 2 and accumulation of intratumor regulatory T cells [22].

We hypothesize that pre-existing type I IFN or IL-6-related inflammation negatively impacts PD-1 blockade and hence therapy effectiveness. Therefore, we tested the effect of IFN-α and IL-6 on the effectiveness of PD-1 blockade in vitro on human T cells in a mixed leukocyte reaction (MLR)*.* In melanoma patient peripheral blood mononuclear cells (PBMCs) we assessed whether the type I IFN score predicts treatment outcome.

## Materials and methods

### Reagents

All reagents used in this study are listed in Table 1.

**Table 1:** Overview of materials

Antibodies						
**Target**	**Label**	**Vendor**	**Clone**	**Catalog#**	**Usage**	**RRID**
PD-1	Unlabeled	Bristol-Myers Squibb	Nivolumab	Lot: ABH9590	10 µg/ml	N.A
CD3	Unlabeled	Life Technologies	OKT3	16–0037-85	0.1 µg/ml	AB_468855
CD3	FITC	Biolegend	OKT3	317306	20x	AB_571907
CD3	AF700	Biolegend	UCHT1	300424	50x	AB_493741
CD4	BV785	Biolegend	RPA-T4	300554	50x	AB_2564382
CD8	BV605	Biolegend	SK1	344742	100x	AB_2566513
CD8a	PerCP-Cy5.5	Biolegend	RPA-T8	301032	100x	AB_893422
PD-1	BV711	BD Biosciences	EH12.1	564017	100x	AB_2738543
CTV	N.A	Life Technologies	N.A	C34557	2 µM	N.A
Fixable Viability Dye	eFluor780	eBioscience	N.A	13539140	2000x	N.A
Fixable Viability Dye	eFluor506	eBioscience	N.A	65–0866-14	1000x	N.A
IFN-γ	PE-Cy7	BD Biosciences	4S.B3	557844	200x	AB_396894
GzmB	APC-Fire750	Biolegend	QA16A02	372210	50x	AB_2728377

Reagents						
**Name**	**Vendor**	**Catalog#**	**Usage**			
CD14 MicroBeads	Miltenyi Biotec	130–050-201	According to manufacturer			
Human pan T cell isolation kit	Miltenyi Biotec	130–096-535	According to manufacturer			
GM-CSF	R&D Systems	215-GM	800 U/ml			
IL-4	R&D Systems	204-IL	500 U/ml			
RPMI 1640 medium	Gibco	12017599	N.A			
Recombinant human IL-6	R&D Systems	7270-IL	0.1 µg/ml			
IFN-α2A	Cell Sciences	CRI003B	100 U/ml			
PMA	Sigma-Aldrich	P8139	20 ng/ml			
Ionomycin	Sigma-Aldrich	I0634	1 mg/ml			
Golgistop	BD Bioscience	554724	1500x			
Brilliant Stain buffer	BD Bioscience	563794	8%			
Normal mouse serum	Bioconnect	88-NM35	2–5%			
Cytofix/Cytoperm solution	BD Bioscience	554722	According to manufacturer			
Human IFNγ Uncoated ELISA kit	Thermo Fisher Scientific	88–7316-88	According to manufacturer			
RNeasy Mini Kit	Qiagen	74106	According to manufacturer			
RNeasy Micro Kit	Qiagen	74004	According to manufacturer			
RNase-Free DNase Set	Qiagen	79254	According to manufacturer			
iScript reverse transcriptase kit	Biorad	1708891	According to manufacturer			
SYBR Select Mastermix	Life Sciences	13266519	According to manufacturer			

Primer sequences						
Gene	Forward Sequence 5’—3’	Reverse Sequence 5’—3’				
*GUSB*	CACCAGGGACCATCCAATACC	GCAGTCCAGCGTAGTTGAAAAA				
*IFITM1*	CCAGCATCCGGACACCACAG	CCCCCAGCACAGCCACCTC				
*Ly6E*	ATCTGTACTGCCTGAAGCCG	GTCACGAGATTCCCAATGCC				
*MX1*	ATCCAGCCACCATTCCAAGG	TGCGATGTCCACTTCGGAAA				
*IFI44L*	CCACCGTCAGTATTTGGAATGT	ATTTCTGTGCTCTCTGGCTT				

Devices and software						
Name	Source					
Clariostar plate reader	BMG LABTECH					
BD Fortessa (4-laser) flow cytometer	BD Bioscience					
BD FACSDiva	BD Bioscience					
FlowJo (v 10.8.1)	BD Bioscience					
QuantStudio 12 K Flex	Life Technologies-Thermo Fisher Scientific					
Prism (v 10.1.2)	Graphpad					
R (v 4.3.1), package survival (v 3.5–5)	R Project					

### Patients and controls

All participants provided written informed consent. Control donors were included in the in-house blood donor service with approval from the University Medical Center (UMC) Utrecht Ethical Committee of Biobanks (TC-bio 18–774) and medical ethical committee (07–125/O). Treatment-naïve melanoma patients were included in the UNraveling Immune Checkpoint Inhibitor induced Toxicity (UNICIT) cohort of the UMC Utrecht. The Biobank Review Committee of the UMC Utrecht gave ethical approval for the UNICIT biobank study (TC-bio 18–123) and granted permission for use of human biospecimens for the present study (TC-bio 19–704) [23].

### Primary cell isolation, culture and differentiation

PBMCs were isolated by Ficoll density gradient. Monocytes were isolated with magnetic activated cell sorting (MACS) human CD14 MicroBeads, and T cells with the MACS human pan T cell isolation kit.

Cells were cultured in RPMI containing 10% bovine serum, 100 U/ml penicillin, 100 μg/ml streptomycin, and 2 mM glutamine (culture medium) at 37 °C with 5% CO_2_ in a humidified cell culture incubator, unless stated otherwise.

Isolated monocytes were differentiated to monocyte-derived dendritic cells (moDCs) with granulocyte–macrophage colony-stimulating factor (GM-CSF) and interleukin (IL)-4 in culture medium for 7 days. On day 4, the culture medium was refreshed with new GM-CSF and IL-4. moDCs were used either directly after differentiation or stored at -80 °C for later use. All reagents used in this study are listed in Table 1.

### Mixed lymphocyte reaction (MLR)

50,000 mismatched control T cells and 10,000 control moDCs were co-cultured in a 96-well plate with or without a monoclonal PD-1 antibody (10 µg/ml, Nivolumab), and with or without anti-CD3 (OKT3, 0.1 µg/ml coated o/n 4 °C or 2 h 37 °C) as a control. Where indicated we added IL-6 (0.1 µg/ml), or IFN-α (100 U/ml) to the system. After 3 and 6 days of MLR, culture cells were harvested for flow cytometry. After 6 days of MLR, we spun down the plates and harvested cell-free supernatant to assess cytokine secretion by ELISA. Cell-free supernatant was frozen down at − 20 °C and thawed when performing ELISAs.

120,000 mismatched melanoma patient-derived PBMCs were co-cultured with 10,000 moDCs from a pool of control donors in a 96-well plate with or without a monoclonal PD-1 antibody (10 µg/ml, Nivolumab), and with or without anti-CD3 (OKT3, 0.1 µg/ml coated o/n 4 °C or 2 h 37 °C) as a control. After 6 days of MLR, we spun down the plates and harvested cell-free supernatant to assess cytokine secretion by ELISA. Cell-free supernatant was frozen down at − 20 °C and thawed when performing ELISAs. All reagents used in this study are listed in Table 1.

### Enzyme-linked immunosorbent assay (ELISA)

IFN-γ secretion was measured from cell-free supernatant using the Human IFNγ Uncoated ELISA kit according to the manufacturer’s protocol. Optical densities (OD) were measured using a Clariostar plate reader. We used Prism to construct a 4-parameter dose response curve based on the standard ODs and extrapolated the unknown concentrations. We used 0.5 × the lower limit of detection for values beneath the standard curve (18). All reagents used in this study are listed in Table 1.

### Fluorescence activated cell sorting (FACS)

#### Proliferation

To assess proliferation, control T cells were labeled with cell trace violet (CTV) before they were co-cultured with moDCs in the MLR. After 3 and 6 days of MLR, cells were stained with a fixable viability dye (eFluor780) and surface stained (CD3-FITC, CD4-BV785, CD8-BV605) for 20 min at 4 °C while aspecific antibody binding was prevented with 2% normal mouse serum [24]. Gating strategy is depicted in SI Fig. 1a.

#### Maximum IFN-γ production

To assess maximum IFN-γ production after 6 days of control MLR, cells were stimulated with Phorbol 12-myristate 13-acetate (PMA) and ionomycin for 4 h at 37 °C. After 30 min, Golgistop was added for the remaining 3.5 h. Cells were stained with a fixable viability dye (eFluor780) and surface stained (CD3-FITC, CD4-BV785, CD8-BV605) for 20 min at 4 °C while FC-receptors were blocked with normal mouse serum, fixed with Cytofix/Cytoperm solution for 30 min at 4 °C, and intracellular stained (IFNγ-PE-Cy7) for 20 min at 4 °C while aspecific antibody binding was prevented with 2% normal mouse serum [24]. Gating strategy is depicted in SI Fig. 1b.

Pre-treatment T cell characteristicsTo assess the pre-treatment T cell characteristics of responders and non-responders melanoma patient-derived PBMCs were both surface and intracellular stained. For the surface staining, cells were stained with a fixable viability dye (eFluor506) and stained (CD3-AF700, CD4-BV785, CD8a-PerCP-Cy5.5, PD-1-BV711) for 20 min at 4 °C while aspecific antibody binding was prevented with 2% normal mouse serum [24]. Gating strategy is depicted in SI Fig. 1c.

For intracellular straining of cytokines, cells were stimulated with PMA and ionomycin for 4 h at 37 °C. After 30 min, Golgistop was added for the remaining 3.5 h. Cells were stained with a fixable viability dye (eFluor506) and surface stained (CD3-AF700, CD4-BV785, CD8a-PerCP-Cy5.5, PD1-BV711) for 20 min at 4 °C while aspecific antibody binding was prevented with 2% normal mouse serum, fixed with Cytofix/Cytoperm solution for 30 min at 4 °C, and intracellular stained (IFNγ-PE-Cy7, GzmB-APC-Fire650) for 20 min at 4 °C while aspecific antibody binding was prevented with 2% normal mouse serum (24). Gating strategy is depicted in SI Fig. 1d.

In antibody mixes that contained two or more Brilliant Violet fluorescent dyes, we used 8% brilliant stain buffer to prevent staining artifacts due to interaction between brilliant violet dyes. All samples were acquired on a BD LSR Fortessa using BD FACSDiva software. Data were analyzed using FlowJo software. All reagents used in this study are listed in Table 1.

### RNA isolation and quantitative real-time PCR (RT-qPCR)

We isolated total RNA from cell lysates using the RNeasy micro/mini kit according to the manufacturer’s protocol and included the optional DNA digestion. cDNA was synthesized using the iScript reverse transcriptase kit according to the manufacturer’s protocol with two exceptions. Firstly, reverse transcription was performed for 40 min instead of 20 min at 46 °C, and secondly, reverse transcriptase inactivation was performed for 5 min instead of 1 min at 95 °C. Gene expression was determined in duplo by RT-qPCR on the QuantStudio 12 k flex using SybrGreen mastermix with specific primer sets and averaged. Relative gene expression (2^ΔCt^) of the averages was normalized using the *GUSB* housekeeping gene and then Z-normalized per gene. Type I IFN score was calculated as the sum of individual z-values of the Type I IFN related genes *Ly6E*, *MX1*, *IFI44L* and *IFITM1*. All reagents used in this study are listed in Table 1.

### Statistics

Statistical analysis was performed in prism and survival analysis in R with package *survival*. Wilcoxon tests were performed for paired, not Gaussian distributed data and Mann–Whitney tests were performed for unpaired, not Gaussian distributed data. For data that passed normality tests, paired or unpaired T-tests were performed. For data that passed log-normality tests, the data were log-transformed before paired or unpaired T-tests were performed. Progression-free survival was assessed with the Kaplan–Meier method and groups were compared by a log-rank test. In all figure legends, we have indicated the statistical test used to determine significance, and the “n” of experiments. Data are considered significant if *p* < 0.05.

## Results

### IFN-α reduces the effectiveness of PD-1 blockade in vitro

**Fig. 1:**
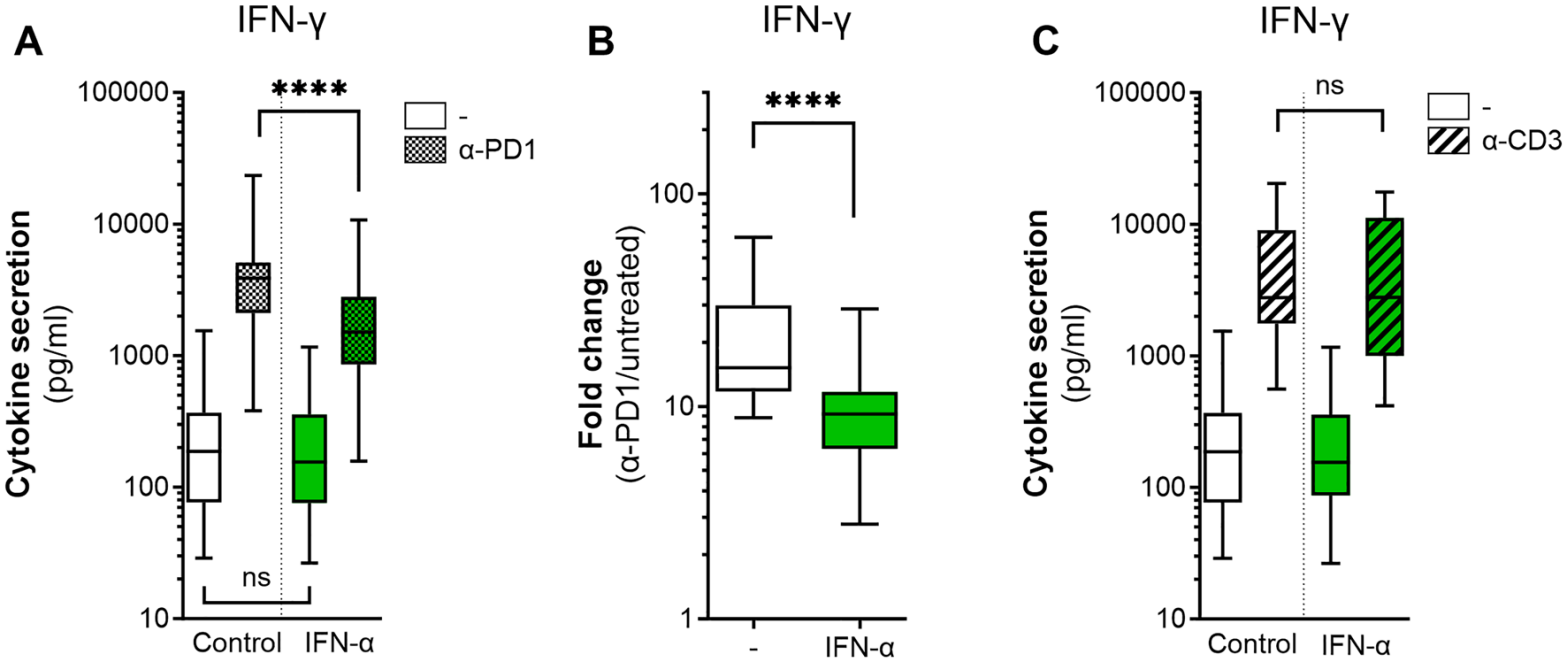
IFN-α reduces IFN-γ secretion in response to PD-1 blockade in vitro **a** IFN-γ secretion measured by ELISA after 6 days of mixed lymphocyte reaction (MLR) with 50,000 mismatched T cells and 10,000 moDCs of control donors, untreated or PD-1 blockade treated (αPD1; 10 µg/ml). The MLR was exposed to medium (control, *n* = 21), or 100 U/ml IFN-α (*n* = 21). **b** Data of Fig. 1a expressed as fold changes of IFN-γ secretion between αPD1 treated and untreated cells, exposed to medium (control, *n* = 21), or 100 U/ml IFN-α (*n* = 21). Fold change = [IFN-γ] in αPD1 treated cells “Fig. 1a”/[IFN-γ] in untreated cells “Fig. 1a”. **c** IFN-γ secretion measured by ELISA after 6 days of mixed lymphocyte reaction (MLR) with 50,000 mismatched T cells and 10,000 moDCs of control donors, untreated (same data as in “Fig. 1a”) or treated with agonistic CD3 antibodies (αCD3; 0.1 µg/ml). The MLR was exposed to medium (control, *n* = 17), or 100 U/ml IFN-α (*n* = 17). **a**–**c** Experiments were performed in duplo or triplo per “n”, which were averaged. Averages are plotted as boxplots with medians and interquartile ranges (IQR). Depicted significance was determined using paired T tests on log transformed data

We set up a mixed lymphocyte reaction (MLR) with healthy donor CD3^+^ T cells and mismatched monocyte-derived DC (moDCs) to study the effect of inflammatory cytokines on the enhancement of T cell IFN-γ secretion by PD-1 blockade. After 6 days of culture, T cells secreted more IFN-γ in the presence of an antagonistic PD-1 antibody (αPD1, Nivolumab) than control-treated T cells (Fig. 1a). Adding exogenous IFN-α reduced IFN-γ secretion in the presence of PD-1 blockade (Fig. 1a), but had no effect on IFN-γ secretion without PD-1 blockade. When analyzing this data as fold change of IFN-γ secretion with vs. without PD-1 blockade, IFN-α reduced the effectiveness of PD-1 blockade by twofold (Fig. 1b). Adding exogenous IL-6, another inflammatory stimulus, reduced IFN-γ secretion by T cells in the MLR in both the absence and presence of PD-1 blocking antibodies (SI Fig. 2a). The fold change of IFN-γ secretion with vs. without PD-1 blockade was therefore similar with and without IL-6 (SI Fig. 2b). We conclude that IL-6 reduces IFN-γ secretion by T cells both in the absence and presence of PD-1 blockade in the MLR, suggesting that IL-6 affects T cell activation in general. Since we observed that IFN-α reduces the effectiveness of PD-1 blockade, in contrast to IL-6, we focused on IFN-α. IFN-α did not affect the intrinsic capacity of T cells to become activated during the MLR, since IFN-α did not reduce the absolute amount of IFN-γ secreted by T cells in the presence of agonistic CD3 antibodies (αCD3) (Fig. 1c). Therefore, we conclude that IFN-α specifically reduces the effectiveness of PD-1 blockade to induce IFN-γ in vitro.

**Fig. 2:**
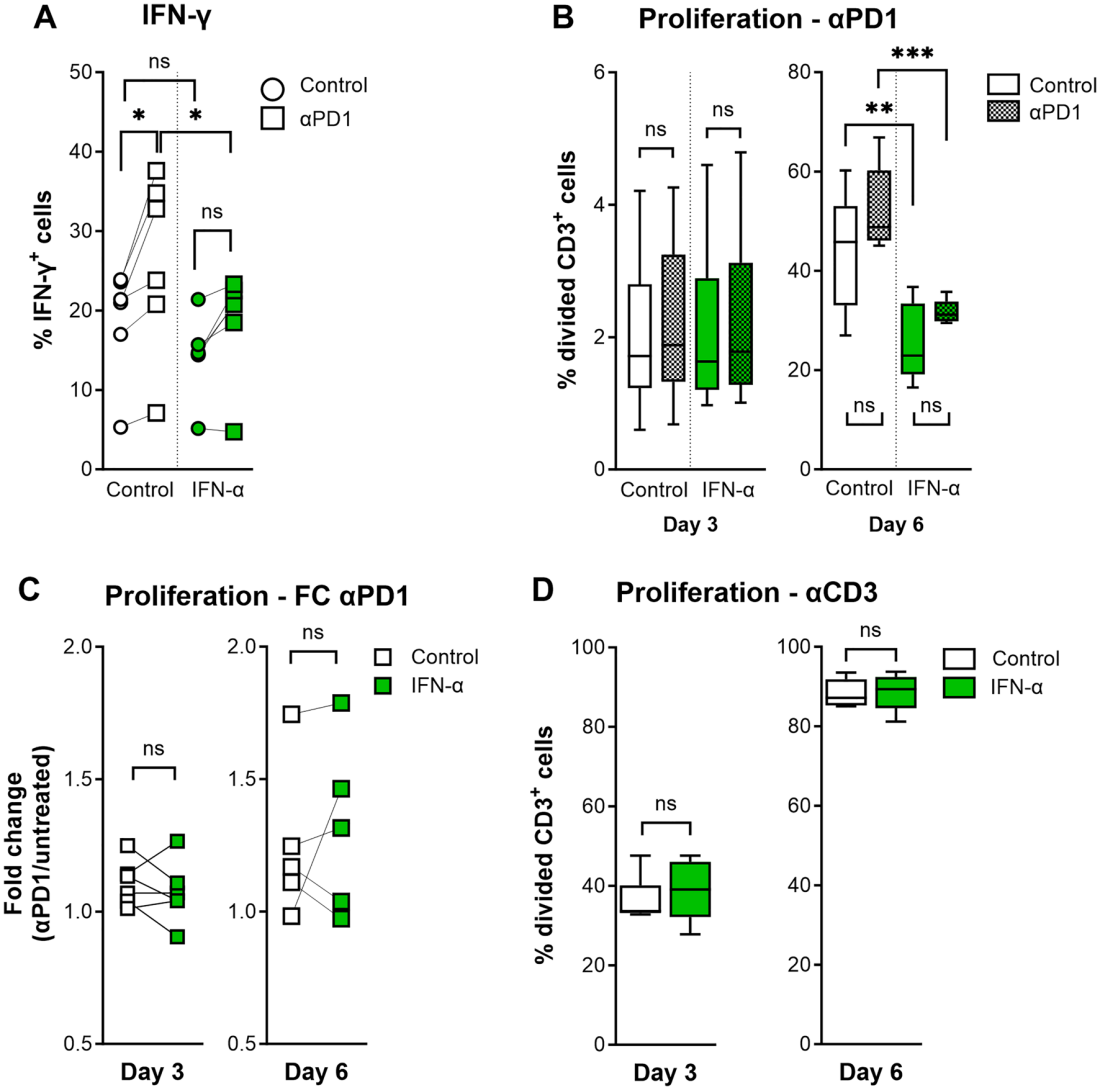
IFN-α interferes with T cell activation in vitro **a** Percentage IFN-γ^+^ cells of 4 h PMA/ionomycin treated alive CD3^+^ cells of control donors after 6 days of MLR (*n* = 6). Experiments were performed in triplo and medians are shown. Wilcoxon tests were used to determine significance. **b** Proliferation of CD3^+^ T cells after 3 (*n* = 6) and 6 days (*n* = 5) of MLR, untreated or αPD1 treated, exposed to medium, or in the presence of exogenous IFN-α. T cells were labeled with CTV before the start of the MLR. Proliferation was measured with flow cytometry as percentage CTV^low^ alive CD3^+^ cells. **c** Fold changes of percentage division between αPD1 treated and untreated cells as measured in Fig. 1b. Fold change = [% CTV^−^ alive CD3^+^ cells] in αPD1 treated cells / [% CTV^−^ alive CD3^+^ cells] in untreated cells. **d** Proliferation of CD3^+^ T cells after 3 (*n* = 6) and 6 days (*n* = 5) of MLR in the presence of agonistic αCD3 antibodies, with or without exogenous IFN-α. T cells were labeled with CTV before the start of the MLR. Proliferation was measured by flow cytometry as percentage CTV^low^ alive CD3^+^ cells. **b**–**d** Experiments of day 3 were performed in triplo per “*n*”, and medians were calculated. Medians are shown (**c**) or plotted as boxplots with medians and IQR (**b and d**). Experiments of day 6 were performed in monoplo or duplo per “*n*”, and means were calculated. Means are shown (**c**) or plotted as boxplots with medians and IQR (**b and d**). Significance was determined using paired T tests

In the absence of exogenous IFN-α, PD-1 blockade increased the percentage of T cells that produced IFN-γ (Fig. 2a). However, adding IFN-α decreased the ability of PD-1 blockade to increase the percentage of T cells that produced IFN-γ, while IFN-α did not change the percentage of T cells that produced IFN-γ in absence of PD-1 blockade (Fig. 2a). We therefore investigated whether IFN-α reduces MLR-induced proliferation of T cells in the presence of PD-1 blockade. We found that exogenous IFN-α reduced the percentage of proliferated cell trace violet (CTV) labeled T cells in both absence and presence of PD-1 blockade at day 6 of the MLR (Fig. 2b, c). In contrast, IFN-α did not influence T cell proliferation induced by agonistic αCD3 (Fig. 2d), suggesting that IFN-α has anti-proliferative effects that are overruled by αCD3 stimulation but not by PD-1 blockade. We conclude that IFN-α reduced MLR-induced proliferation independent of PD-1 blockade and reduced the frequency of IFN-γ-expressing cells in the presence of PD-1 blockade.

### Immune characteristics of cohort of melanoma patients

To address whether the MLR was predictive for PD-1 blockade treatment outcome in patients, we used PBMCs from melanoma patients enrolled in the Unraveling Immune Checkpoint inhibitor induced toxicity (UNICIT) cohort in the UMC Utrecht [23]. PBMCs were collected and cryopreserved prior to the start of PD-1 blockade treatment. We selected 24 patients with irresectable stage III or stage IV melanoma that received single-agent PD-1 blockade treatment and assessed their best overall response per RECIST 1.1 [25]. Two patients were excluded because of too low recovery of cells after thawing. The remaining 22 patients had a median age of 73.5 years, and 45% was male (Table 2). Three patients reached complete response, 9 partial response and 10 had progressive disease. We considered patients who reached complete response or partial response as clinical responders (n = 12), and patients with progressive disease as non-responders (n = 10). Responders and non-responders were of similar age and sex, had a similar range of lactate dehydrogenase and C-reactive protein concentrations and similar immune cell counts (Table 2). In addition, we found no overt differences in PBMC composition between responders and non-responders (Table 2). PBMCs from responders and non-responders were similar in CD4^+^/CD8^+^ T ratio, PD-1 expression on total CD3^+^ T cells, CD4^+^ and on CD8^+^ T cells (SI Fig. 3a, b). The intrinsic capacity of T cells in these PBMCs to produce IFN-γ and Granzyme B upon stimulation with PMA/ionomycin was similar between responders and non-responders, for both CD4^+^ and CD8^+^ T cells (SI Fig. 3c, d).

**Table 2:** Treatment-naïve melanoma patient’s characteristics

	All (*n* = 22)	Responders (*n* = 12)	Non-responders (*n* = 10)	*P*-value for difference R vs. NR
Median age, years (IQR)	74 (66–79)	77 (61–81)	70 (63–77)	*p* = 0.1735
Female (%)	12 (55)	6 (50)	6 (60)	
Median LDH (U/L) (IQR)	205 (189–232)	221 (203–233)	193 (182–217)	*p* = 0.1033
Median CRP (mg/L) (IQR)	1.4 (0.93–6.6)	1.7 (1.0–7.8)	1.2 (0.5–6.9)	*p* = 0.4823
Tumor stage				
III unresectable	5	3	2	
IV	17	9	8	
BOR				
CR	3	3	–	
PR	9	9	–	
PD	10	-	10	
Median leukocytes (*10^9/L) (IQR)	7.8 (6.6–11)	7.8 (7.2–11)	7.7 (5.4–11)	*p* = 0.4668
Median lymphocytes (*10^9/L) (IQR)	2.1 (1.7–2.4)	2.2 (1.6–2.5)	2.1 (1.7–2.4)	*p* = 0.5933
Median monocytes (*10^9/L) (IQR)	0.64 (0.53–0.89)	0.67 (0.53–0.8)	0.61 (0.49–0.94)	*p* = 0.8335
Median neutrophils (*10^9/L) (IQR)	4.8 (3.7–7.2)	4.8 (3.8–7.0)	4.8 (2.8–7.3)	*p* = 0.5824

Mann–Whitney test between responders and non-responders did not show significant differences between groups for age, sex, LDH, CRP, leukocytes, lymphocytes, monocytes and neutrophils

*LDH*, Lactate dehydrogenase; *CRP*, C-reactive protein; *BOR*, Best overall response; *CR*, Complete response; *PR*, partial response; *PD*, Progressive disease

### Type I IFN score does not correlate with therapy response

**Fig. 3:**
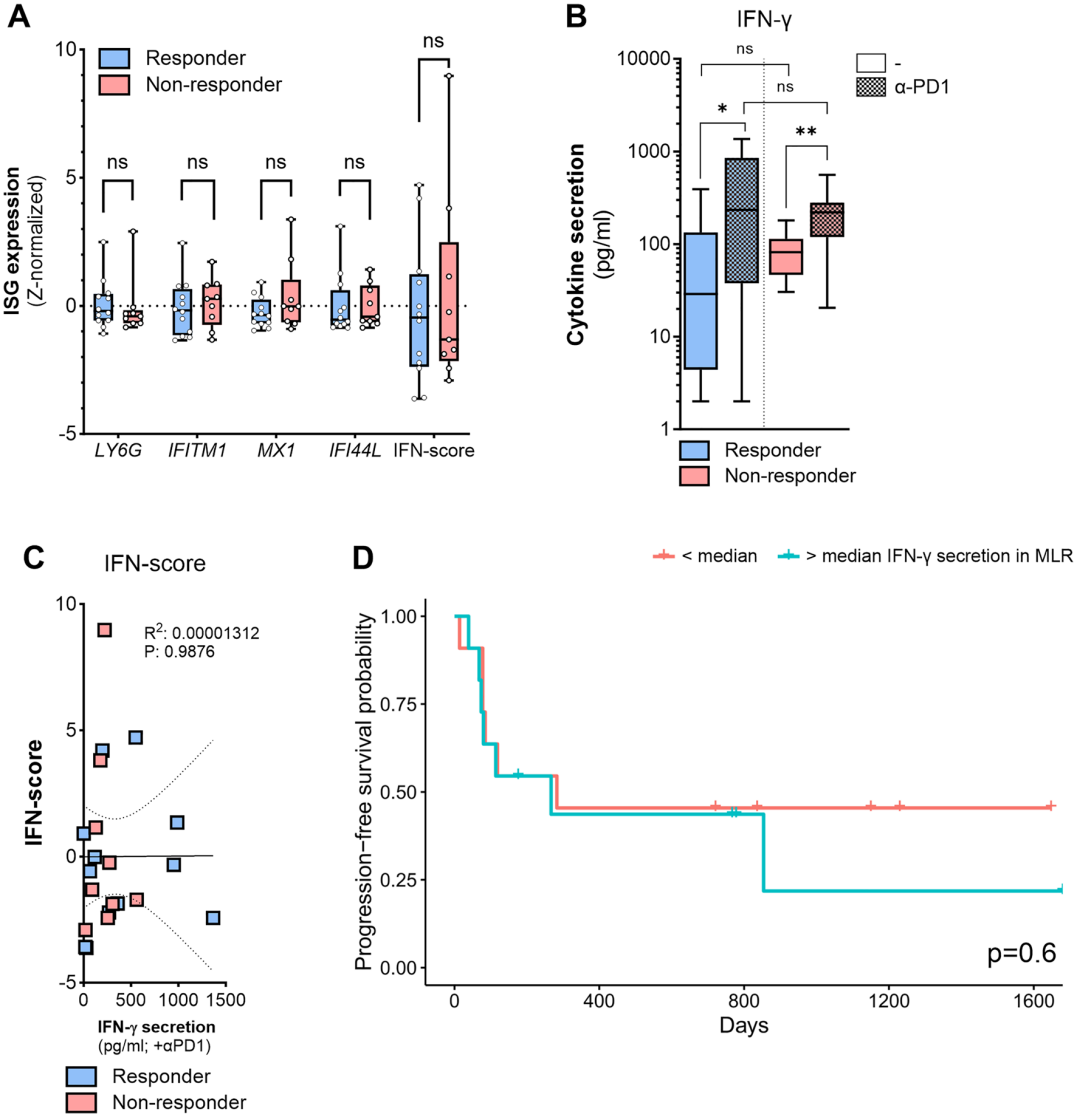
An in vitro MLR with PBMCs is not predictive for PD-1 treatment response. **a** Z-normalized mRNA expression of interferon stimulated genes (ISG) *Ly6E*, *MX1*, *IFI44L* and *IFITM1* in treatment-naïve PBMCs of clinical responders (*n* = 12) and non-responders (*n* = 9). Type I IFN score = sum of z-value of *Ly6E*, *MX1*, *IFI44L* and *IFITM1*. Significance determined with unpaired T tests. **b** MLR with untreated or αPD1 treated cells (10 µg/ml) with treatment-naïve PBMCs from responders (*n* = 12) or non-responders (*n* = 10). Experiments were performed in triplo per “n”, and medians were calculated. Medians are plotted as boxplots with medians and IQR. Significance determined with T tests. **c** Correlation of type I IFN score “Fig. 3a with IFN-γ secretion induced by PD-1 blockade “Fig. 3b” (*n* = 21). Significance determined with simple linear regression. **d** Kaplan–Meier curve of progression-free survival in patients with IFN-γ secretion “Fig. 3b” above or below median upon PD-1 blockade (10 µg/ml) in the MLR (*n* = 22). Comparison by log-rank test

We hypothesized that type I IFN exposure in vivo prior to therapy weakens therapy response in patients. The type I IFN score is a measure for in vivo IFN exposure frequently used in studies of autoimmunity [26]. We used mRNA expression of the type I IFN-responsive genes *Ly6E*, *MX1*, *IFI44L* and *IFITM1* to determine the pre-treatment type I IFN score in the PBMCs from the melanoma patients in our study. The pre-treatment type I IFN score did not differ between responders and non-responders (Fig. 3a). Thus, pretreatment type I IFN exposure based on these type I IFN-responsive genes did not determine clinical response to PD-1 blockade therapy in our cohort.

### An in vitro MLR with PBMCs is not predictive for PD-1 treatment response

We performed the MLR with pooled moDCs from 3 donors to minimize variation introduced by the moDCs. We used PBMCs, and not isolated T cells, because T cells isolated from frozen PBMCs were not viable after 6 days of culture in MLR (data not shown). αCD3-stimulation resulted in similar amounts of IFN-γ produced by PBMCs from responders and non-responders (SI Fig. 3e). In contrast to MLRs with isolated control T cells, in the MLR with control PBMCs IFN-α did not reduce the effectiveness of PD-1 blockade (SI Fig. 3f). However, we reasoned that in vivo exposure to inflammation could have functional consequences in the MLR. Hence, we performed the MLR with PBMCs from melanoma patients without exogenous inflammatory stimuli. After 6 days of MLR culture, PBMCs of both responders and non-responders increased IFN-γ secretion upon PD-1 blockade (Fig. 3b), but there was no obvious difference in IFN-γ secretion between responders and non-responders (Fig. 3b). In addition, the increase of IFN-γ secretion by PD-1 blockade in the MLR did not correlate with the type I IFN score in responders and non-responders (Fig. 3c), neither did IFN-γ secretion by PD-1 blockade correlate with PD-1 expression on CD3^+^ T cells (SI Fig. 4. We found no correlation between progression-free survival of melanoma patients in our cohort and the absolute amount of IFN-γ secretion in presence of PD-1 blockade in the PBMC MLR (Fig. 3d). Together, these data suggest that IFN-γ secretion in the MLR with PBMCs is not predictive for patient responses.

## Discussion

In large patient groups, there is a negative correlation between the concentration of IL-6 or CRP in serum of patients, and the outcome of PD-1 blockade treatment [16–21]. Similarly, type I IFN signaling in the tumor is associated with treatment resistance [22]. The in vitro data with isolated CD3^+^ T cell MLRs recapitulated these findings. However, the MLR with patient PBMCs, or the type I IFN score in pre-treatment PBMCs in our cohort did not correlate significantly with clinical treatment response. Hence, our data show that PD-1 blockade in vitro is sensitive to IFN-α, but that the effect of IFN type I on PD-1 blockade is not present or not detectible in the peripheral blood T cells of melanoma patients. Hence, we conclude that an MLR is not suitable to predict per-patient responses.

In our cohort, we did not find the previously reported correlation of CRP concentrations with clinical therapy response [16–20]. This could be due to the fact that by chance, only patients with moderately elevated CRP were included. It is therefore possible that results would have been different if we would have included more patients with high CRP. We did not study the effect of inflammation on moDCs during the MLR, while IFN-α and IL-6 also have modulating effects on moDCs [27–29]. Hence, we cannot exclude that IL-6 or IFN-α change the signals provided by moDCs to activate T cells. In our assays, we compared PD-1 blockade with medium control and therefore cannot exclude that the effect IFN-α has on the effectiveness of PD-1 blockade is (partly) Fc-receptor mediated. Patient MLRs were performed with PBMCs and not with isolated CD3^+^ T cells because T cells isolated from frozen PBMCs were not viable after 6 days of MLR. In MLR with control PBMCs, IFN-α did not reduce the effectiveness of PD-1 blockade, which prevented us from studying the effects of exogenous IFN-α on PD-1 responses in patient samples. Direct proinflammatory effects of IFN-α on monocytes [30–32] in the PBMCs could negate the negative effect IFN-α has on moDCs or CD3^+^ T cells in the MLR.

In both absence and presence of PD-1 blockade, exogenous IL-6 reduced IFN-γ secretion by T cells, but did not alter the fold change of IFN-γ that PD-1 blockade induced in the MLR assays. This suggests that IL-6 affects T cell activation in general and that, as suggested by others, IL-6 blockade therapy may be a valid addon strategy to improve immune checkpoint blockade therapy [33–35].

In the absence of PD-1 blockade, exogenous IFN-α had differential effects on T cell proliferation in the MLR: while we found that IFN-γ secretion was not affected, proliferation was inhibited by exogenous IFN-α. In contrast, in the presence of PD-1 blockade, exogenous IFN-α reduced both IFN-γ secretion and proliferation in the MLR. IFN-α is a known inhibitor of proliferation [36–38] and hence potentially limits the number of IFN-γ secreting T cells. On the other hand, IFN-α promotes T cell skewing toward an IFN-γ secreting Th-1 phenotype [39–41] and induces expression of multiple inhibitory immune receptors on T cells [42–44]. Possibly the balance between TCR-signaling and IFN-α receptor signaling results in differential outcomes regarding IFN-γ secretion in the MLR, specifically reducing the effect of PD-1 blockade on IFN-γ secretion.

In summary, we conclude that an MLR with CD3^+^ T cells recapitulates the correlation of increased IFN-α and IL-6 with reduced T cell responses in melanoma patients. However, results from our MLR with patient’s PBMCs and the type I IFN score did not correlate with their individual clinical response to PD-1 blockade treatment.

### Supplementary Information

Below is the link to the electronic supplementary material.

Supplementary file1 (PDF 841 KB)

## Data Availability

All data generated during and/or analyzed during the current study is available within the manuscript, or supplementary.

## References

[1] Iwai Y, Terawaki S, Honjo T (2005) PD-1 blockade inhibits hematogenous spread of poorly immunogenic tumor cells by enhanced recruitment of effector T cells. Int Immunol 17(2):133–144 15611321 10.1093/intimm/dxh194

[2] Sharma P, Allison JP (2015) The future of immune checkpoint therapy. Science 348(6230):56–61 25838373 10.1126/science.aaa8172

[3] Sharpe AH, Pauken KE (2018) The diverse functions of the PD1 inhibitory pathway. Nat Rev Immunol 18(3):153–167 28990585 10.1038/nri.2017.108

[4] Suijkerbuijk KPM, van Eijs MJM, van Wijk F, Eggermont AMM (2024) Clinical and translational attributes of immune-related adverse events. Nat Cancer 5(4):557–571 38360861 10.1038/s43018-024-00730-3

[5] Das S, Johnson DB (2019) Immune-related adverse events and anti-tumor efficacy of immune checkpoint inhibitors. J Immunother Cancer 7(1):306 31730012 10.1186/s40425-019-0805-8 PMC6858629

[6] Bai X, Shoushtari AN, Betof Warner A, Si L, Tang B, Cui C et al (2022) Benefit and toxicity of programmed death-1 blockade vary by ethnicity in patients with advanced melanoma: an international multicentre observational study. Br J Dermatol 187(3):401–410 35293617 10.1111/bjd.21241

[7] Freeman GJ, Long AJ, Iwai Y, Bourque K, Chernova T, Nishimura H et al (2000) Engagement of the PD-1 immunoinhibitory receptor by a novel B7 family member leads to negative regulation of lymphocyte activation. J Exp Med 192(7):1027–1034 11015443 10.1084/jem.192.7.1027 PMC2193311

[8] Hui E, Cheung J, Zhu J, Su X, Taylor MJ, Wallweber HA et al (2017) T cell costimulatory receptor CD28 is a primary target for PD-1-mediated inhibition. Science 355(6332):1428–1433 28280247 10.1126/science.aaf1292 PMC6286077

[9] Francisco LM, Sage PT, Sharpe AH (2010) The PD-1 pathway in tolerance and autoimmunity. Immunol Rev 236:219–242 20636820 10.1111/j.1600-065X.2010.00923.x PMC2919275

[10] Zou W, Chen L (2008) Inhibitory B7-family molecules in the tumour microenvironment. Nat Rev Immunol 8(6):467–477 18500231 10.1038/nri2326

[11] Arasanz H, Gato-Canas M, Zuazo M, Ibanez-Vea M, Breckpot K, Kochan G, Escors D (2017) PD1 signal transduction pathways in T cells. Oncotarget 8(31):51936–51945 28881701 10.18632/oncotarget.17232 PMC5584302

[12] Yokosuka T, Takamatsu M, Kobayashi-Imanishi W, Hashimoto-Tane A, Azuma M, Saito T (2012) Programmed cell death 1 forms negative costimulatory microclusters that directly inhibit T cell receptor signaling by recruiting phosphatase SHP2. J Exp Med 209(6):1201–1217 22641383 10.1084/jem.20112741 PMC3371732

[13] Veillette A (2010) SLAM-family receptors: immune regulators with or without SAP-family adaptors. Cold Spring Harb Perspect Biol 2(3):a002469 20300214 10.1101/cshperspect.a002469 PMC2829957

[14] Peled M, Tocheva AS, Sandigursky S, Nayak S, Philips EA, Nichols KE et al (2018) Affinity purification mass spectrometry analysis of PD-1 uncovers SAP as a new checkpoint inhibitor. Proc Natl Acad Sci USA 115(3):E468–E477 29282323 10.1073/pnas.1710437115 PMC5776966

[15] Endt J, Eissmann P, Hoffmann SC, Meinke S, Giese T, Watzl C (2007) Modulation of 2B4 (CD244) activity and regulated SAP expression in human NK cells. Eur J Immunol 37(1):193–198 17171759 10.1002/eji.200636341

[16] Laino AS, Woods D, Vassallo M, Qian X, Tang H, Wind-Rotolo M, Weber J (2020) Serum interleukin-6 and C-reactive protein are associated with survival in melanoma patients receiving immune checkpoint inhibition. J Immunother Cancer. 8(1):e000842 32581042 10.1136/jitc-2020-000842 PMC7312339

[17] Mirjacic Martinovic K, Vuletic A, Tisma Miletic N, Matkovic S, Gavrilovic D, Ninkovic A et al (2023) Circulating IL-6 is associated with disease progression in BRAFwt metastatic melanoma patients receiving anti-PD-1 therapy. J Clin Pathol 77(5):343–351 10.1136/jcp-2022-208615 36754615

[18] Iivanainen S, Ahvonen J, Knuuttila A, Tiainen S, Koivunen JP (2019) Elevated CRP levels indicate poor progression-free and overall survival on cancer patients treated with PD-1 inhibitors. ESMO Open 4(4):e000531 31555483 10.1136/esmoopen-2019-000531 PMC6735669

[19] Weber JS, Sznol M, Sullivan RJ, Blackmon S, Boland G, Kluger HM et al (2018) A serum protein signature associated with outcome after Anti-PD-1 therapy in metastatic melanoma. Cancer Immunol Res 6(1):79–86 29208646 10.1158/2326-6066.CIR-17-0412

[20] Yoshida T, Ichikawa J, Giuroiu I, Laino AS, Hao Y, Krogsgaard M et al (2020) C reactive protein impairs adaptive immunity in immune cells of patients with melanoma. J Immunother Cancer. 8(1):e000234 32303612 10.1136/jitc-2019-000234 PMC7204799

[21] Wang Y, Ramachandran V, Sui D, Xu K, Haydu LE, Fang S et al (2022) Evaluation of plasma IL-6 in patients with melanoma as a prognostic and checkpoint immunotherapy predictive biomarker. J Invest Dermatol 142(7):2046–2049 34952092 10.1016/j.jid.2021.12.012 PMC9209587

[22] Jacquelot N, Yamazaki T, Roberti MP, Duong CPM, Andrews MC, Verlingue L et al (2019) Sustained Type I interferon signaling as a mechanism of resistance to PD-1 blockade. Cell Res 29(10):846–861 31481761 10.1038/s41422-019-0224-x PMC6796942

[23] van Eijs MJM, Verheijden RJ, van der Wees SA, Nierkens S, van Lindert ASR, Suijkerbuijk KPM et al (2023) Toxicity-specific peripheral blood T and B cell dynamics in anti-PD-1 and combined immune checkpoint inhibition. Cancer Immunol Immunother 72(12):4049–4064 37794264 10.1007/s00262-023-03541-0 PMC10700442

[24] Andersen MN, Al-Karradi SN, Kragstrup TW, Hokland M (2016) Elimination of erroneous results in flow cytometry caused by antibody binding to Fc receptors on human monocytes and macrophages. Cytometry A 89(11):1001–1009 27731950 10.1002/cyto.a.22995

[25] Eisenhauer EA, Therasse P, Bogaerts J, Schwartz LH, Sargent D, Ford R et al (2009) New response evaluation criteria in solid tumours: revised RECIST guideline (version 1.1). Eur J Cancer 45(2):228–247 19097774 10.1016/j.ejca.2008.10.026

[26] Tsokos GC, Lo MS, Costa Reis P, Sullivan KE (2016) New insights into the immunopathogenesis of systemic lupus erythematosus. Nat Rev Rheumatol 12(12):716–730 27872476 10.1038/nrrheum.2016.186

[27] Beyranvand Nejad E, Labrie C, van Elsas MJ, Kleinovink JW, Mittrucker HW, Franken K et al (2021) IL-6 signaling in macrophages is required for immunotherapy-driven regression of tumors. J Immunother Cancer 9(4):e002460 33879600 10.1136/jitc-2021-002460 PMC8061866

[28] Riegel K, Yurugi H, Schloder J, Jonuleit H, Kaulich M, Kirschner F et al (2021) ERK5 modulates IL-6 secretion and contributes to tumor-induced immune suppression. Cell Death Dis 12(11):969 34671021 10.1038/s41419-021-04257-8 PMC8528934

[29] Lapenta C, Gabriele L, Santini SM (2020) IFN-alpha-mediated differentiation of dendritic cells for cancer immunotherapy: advances and perspectives. Vaccines (Basel). 8(4):617 33086492 10.3390/vaccines8040617 PMC7711454

[30] Gerrard TL, Siegel JP, Dyer DR, Zoon KC (1987) Differential effects of interferon-alpha and interferon-gamma on interleukin 1 secretion by monocytes. J Immunol 138(8):2535–2540 3104469

[31] Corssmit EP, Heijligenberg R, Hack CE, Endert E, Sauerwein HP, Romijn JA (1997) Effects of interferon-alpha (IFN-alpha) administration on leucocytes in healthy humans. Clin Exp Immunol 107(2):359–363 9030876 10.1111/j.1365-2249.1997.269-ce1161.x PMC1904568

[32] Lee PY, Li Y, Kumagai Y, Xu Y, Weinstein JS, Kellner ES et al (2009) Type I interferon modulates monocyte recruitment and maturation in chronic inflammation. Am J Pathol 175(5):2023–2033 19808647 10.2353/ajpath.2009.090328 PMC2774066

[33] Li W, Wu Z, Meng W, Zhang C, Cheng M, Chen Y et al (2022) Blockade of IL-6 inhibits tumor immune evasion and improves anti-PD-1 immunotherapy. Cytokine 158:155976 35921790 10.1016/j.cyto.2022.155976

[34] Tsukamoto H, Fujieda K, Miyashita A, Fukushima S, Ikeda T, Kubo Y et al (2018) Combined blockade of IL6 and PD-1/PD-L1 signaling abrogates mutual regulation of their immunosuppressive effects in the tumor microenvironment. Cancer Res 78(17):5011–5022 29967259 10.1158/0008-5472.CAN-18-0118

[35] Hailemichael Y, Johnson DH, Abdel-Wahab N, Foo WC, Bentebibel SE, Daher M et al (2022) Interleukin-6 blockade abrogates immunotherapy toxicity and promotes tumor immunity. Cancer Cell 40(5):509–523 35537412 10.1016/j.ccell.2022.04.004 PMC9221568

[36] Erickson S, Sangfelt O, Castro J, Heyman M, Einhorn S, Grander D (1999) Interferon-alpha inhibits proliferation in human T lymphocytes by abrogation of interleukin 2-induced changes in cell cycle-regulatory proteins. Cell Growth Differ 10(8):575–582 10470857

[37] Mehrotra A, D’Angelo JA, Romney-Vanterpool A, Chu T, Bertoletti A, Janssen HLA, Gehring AJ (2020) IFN-alpha suppresses myeloid cytokine production, impairing IL-12 production and the ability to support T-cell proliferation. J Infect Dis 222(1):148–157 32049318 10.1093/infdis/jiaa064

[38] Chae F, Oleszak E, Fox FE, Trotta P, Mele CA, Hawrylko E, Platsoucas CD (1988) Differential effects of human alpha and gamma interferon on mixed lymphocyte culture and on T-cell-mediated cytotoxicity. Inhibition of proliferation but not of IL-2 production by alpha interferons. Int Arch Allergy Appl Immunol 86(4):361–369 2970436 10.1159/000234620

[39] Huber JP, Farrar JD (2011) Regulation of effector and memory T-cell functions by type I interferon. Immunology 132(4):466–474 21320124 10.1111/j.1365-2567.2011.03412.x PMC3075500

[40] Shibuya H, Hirohata S (2005) Differential effects of IFN-alpha on the expression of various TH2 cytokines in human CD4+ T cells. J Allergy Clin Immunol 116(1):205–212 15990796 10.1016/j.jaci.2005.03.016

[41] Brassard DL, Grace MJ, Bordens RW (2002) Interferon-alpha as an immunotherapeutic protein. J Leukoc Biol 71(4):565–581 11927642

[42] Hafler D, Sumida T, Dulberg S, Schupp J, Stillwell H, Axisa PP et al (2021) Type I Interferon Transcriptional Network Regulates Expression of Coinhibitory Receptors in Human T cells. Res Sq. 10.21203/rs.3.rs-133494/v1 10.1038/s41590-022-01152-y PMC8989655 35301508

[43] Stecher C, Battin C, Leitner J, Zettl M, Grabmeier-Pfistershammer K, Holler C et al (2017) PD-1 blockade promotes emerging checkpoint inhibitors in enhancing T cell responses to allogeneic dendritic cells. Front Immunol 8:572 28588576 10.3389/fimmu.2017.00572 PMC5439058

[44] Sumida TS, Dulberg S, Schupp JC, Lincoln MR, Stillwell HA, Axisa PP et al (2022) Type I interferon transcriptional network regulates expression of coinhibitory receptors in human T cells. Nat Immunol 23(4):632–642 35301508 10.1038/s41590-022-01152-y PMC8989655

