# Supplementary material for: In vitro T cell responses to PD-1 blockade are reduced by IFN-α but do not predict therapy response in melanoma patients
Source: Cancer Immunol Immunother. 2024 Jul 5;73(9):181. doi: 10.1007/s00262-024-03760-z (PMC11226572; doi:10.1007/s00262-024-03760-z)
Supplement: Supplementary file 1 — Supplementary file1 (PDF 841 KB) [file 262_2024_3760_MOESM1_ESM.pdf]

Supplementary Information

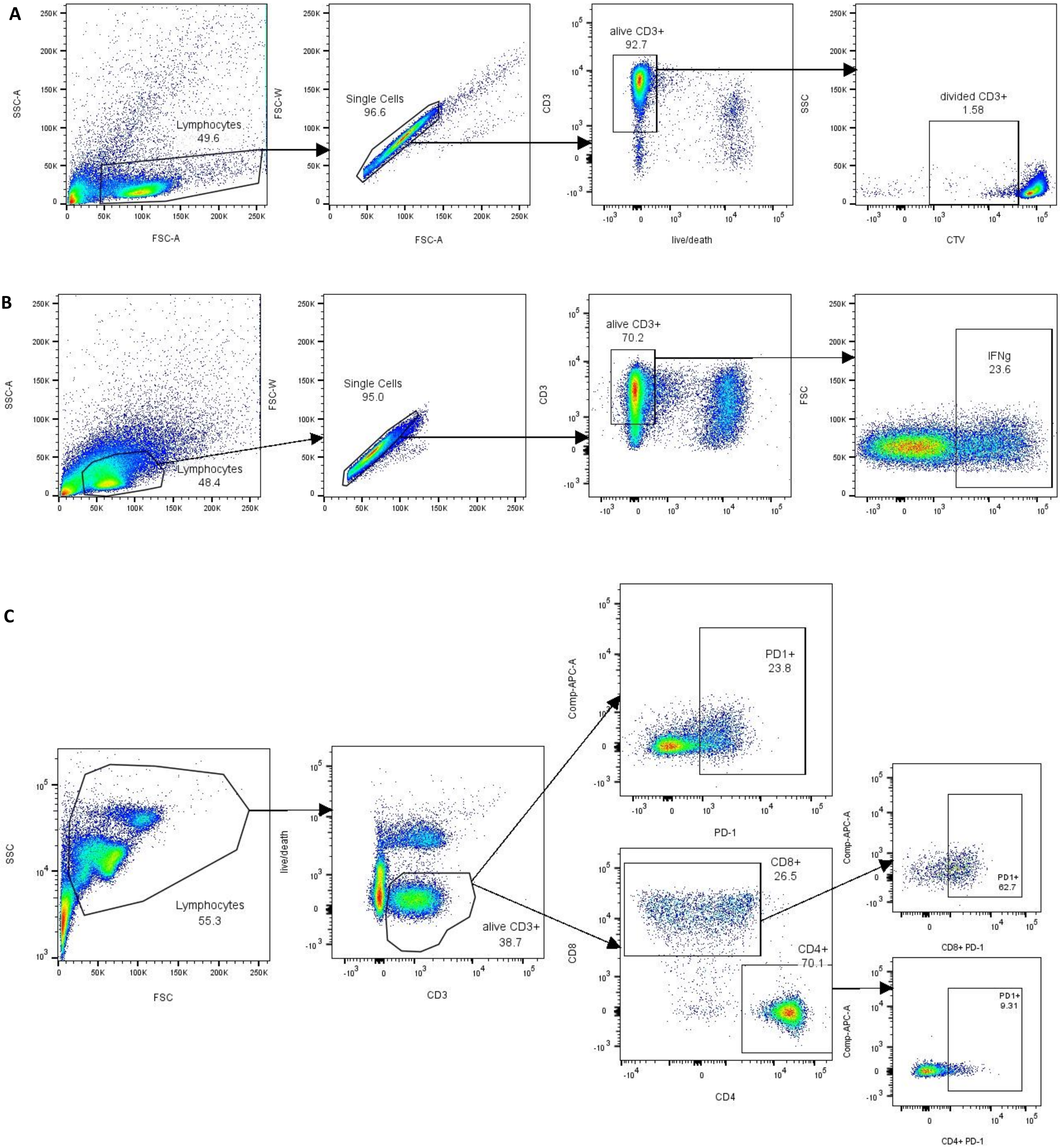

D

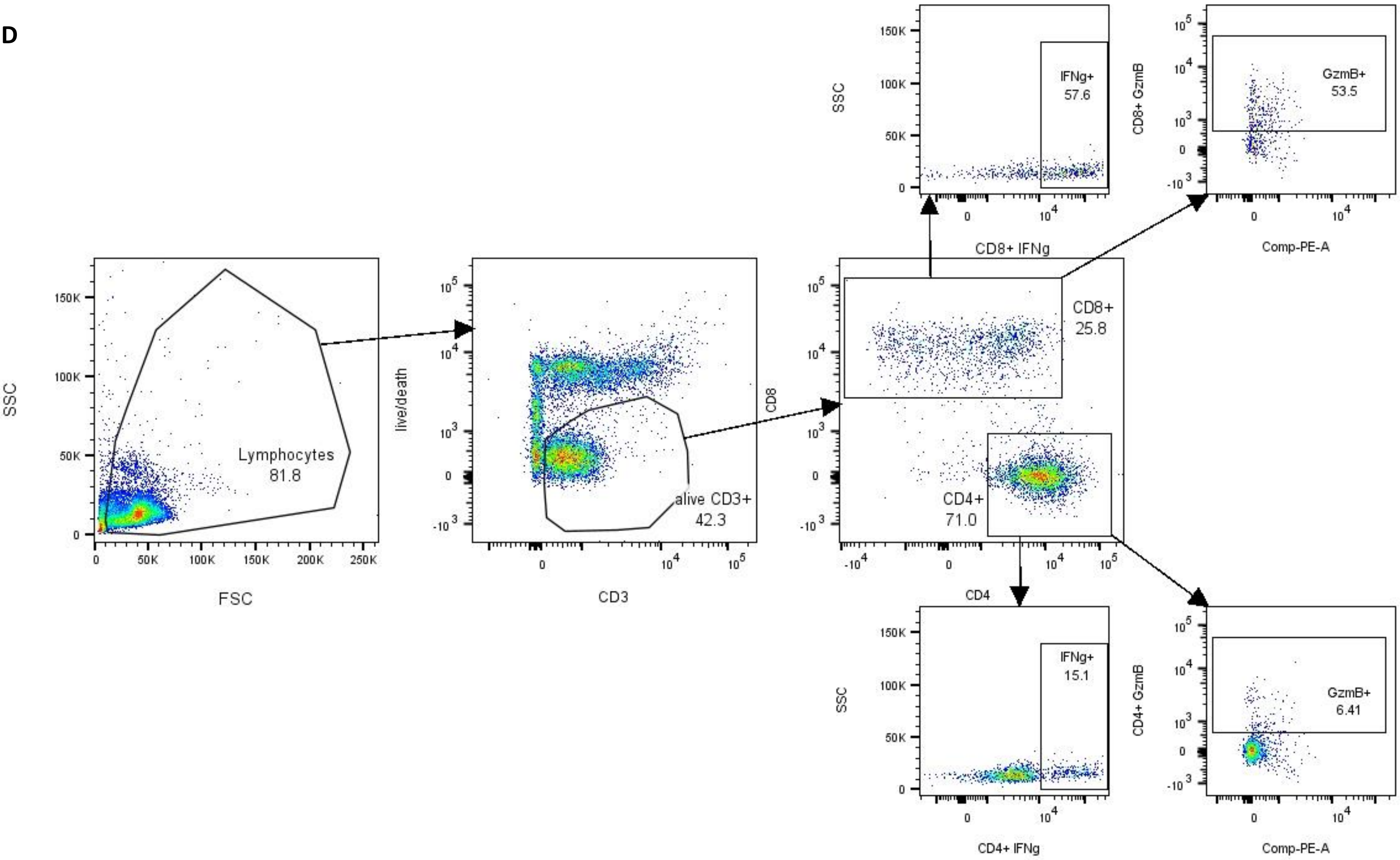

SI Fig.1 Gating strategies for FACS experiments

a) Gating strategy to determine the percentage of divided $CD3^+$ T cells after 3 and 6 days of MLR. Alive $CD3^+$ single cell T cells were gated for division based on CTV staining.

b) Gating strategy to determine maximum IFN-γ production after 6 days of control MLR. Alive $CD3^+$ single cell T cells were gated for IFN-γ expression.

c) Gating strategy of surface-stained melanoma patient derived PBMCs to determine pre-treatment T cell characteristics of responders and non-responders. Alive $CD3^+$ T cells were gated for PD-1, CD4 and CD8 expression. $CD3^+CD4^+$ and $CD3^+CD8^+$ T cells were subsequently gated for PD-1 expression.

d) Gating strategy of intracellular stained melanoma patient derived PBMCs to determine pre-treatment T cell characteristics of responders and non-responders. Alive $CD3^+$ T cells were gated for CD4 and CD8 expression. $CD3^+CD4^+$ and $CD3^+CD8^+$ T cells were subsequently gated for IFN-γ and GzmB expression.

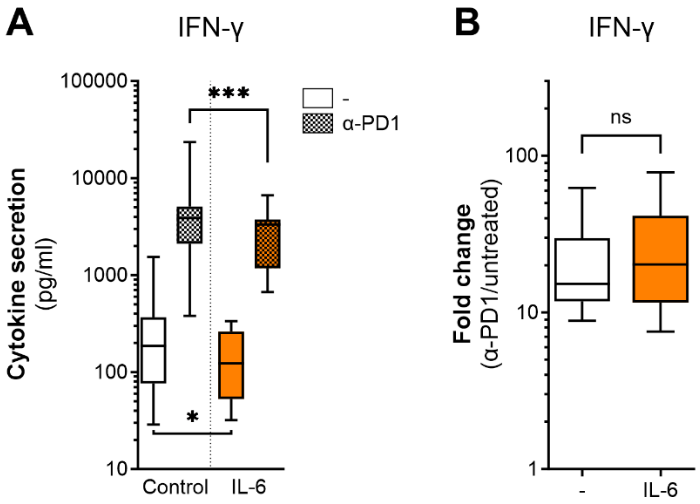

SI Fig.2 IL-6 reduces IFN-γ secretion by T cells both in the absence and presence of PD-1 blockade *in vitro*

a) IFN-γ secretion measured by ELISA after 6 days of mixed lymphocyte reaction (MLR) with 50,000 mismatched T cells and 10,000 moDCs of control donors, untreated or PD-1 blockade treated (αPD1; 10 µg/ml). The MLR was exposed to medium (control, n=21) "Fig.1a", or 0.1 µg/ml IL-6 (n=8).

b) Data of SI Fig.2a expressed as fold changes of IFN-γ secretion between αPD1 treated and untreated cells, exposed to medium (control, n=21), or 0.1 µg/ml IL-6. Fold change = [IFN-γ] in αPD1 treated cells (SI Fig.2a) / [IFN-γ] in untreated cells (SI Fig.2a).

a-b) Experiments were performed in duplo per "n", which were averaged. Averages are plotted as boxplots with medians and interquartile ranges (IQR). Depicted significance was determined using paired T tests on Log transformed data.

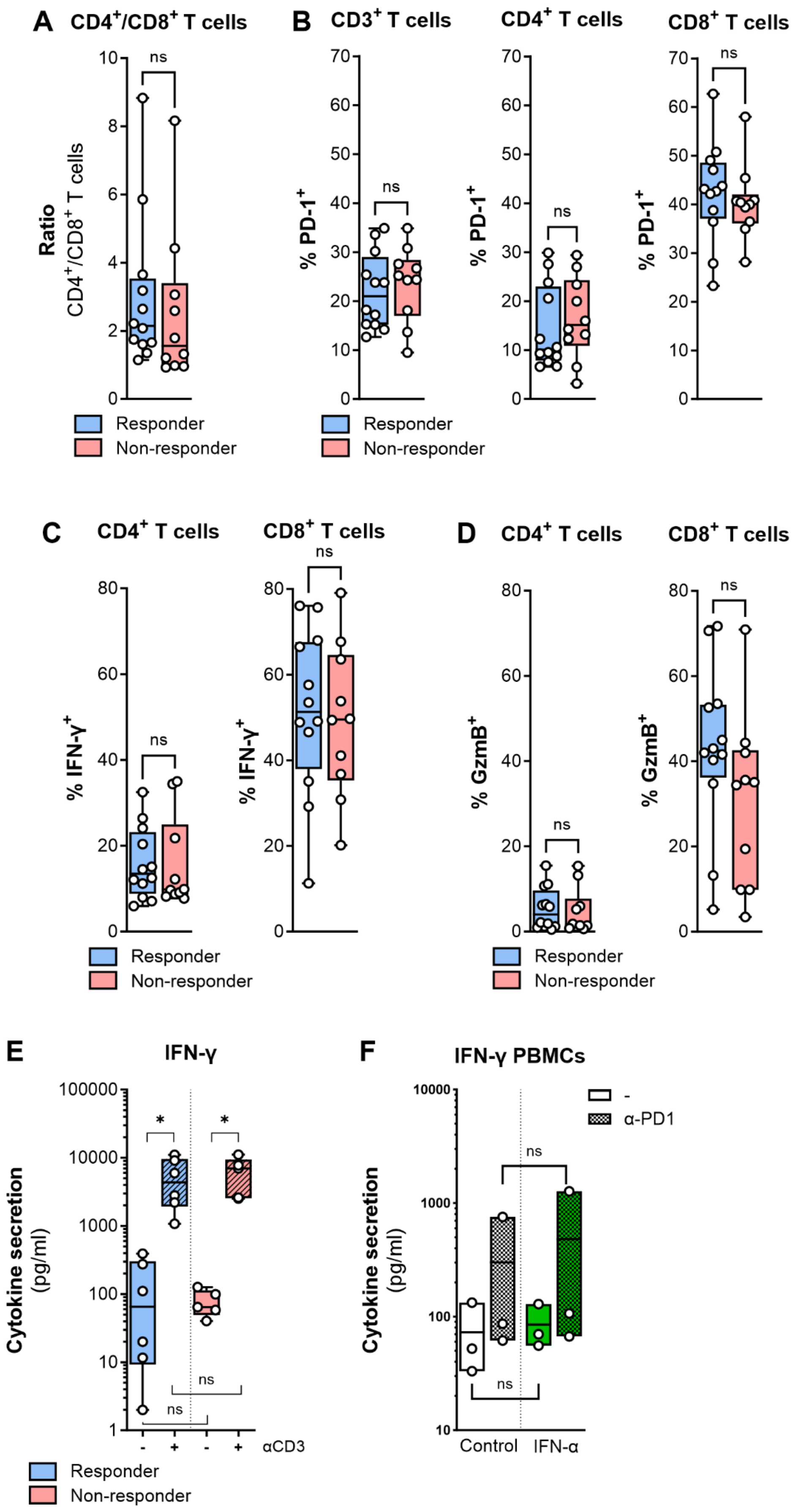

A CD4+/CD8+ T cells
Ratio CD4+/CD8+ T cells
ns
Responder
Non-responder
B CD3+ T cells
CD4+ T cells
CD8+ T cells
% PD-1+
ns
Responder
Non-responder
C CD4+ T cells
CD8+ T cells
% IFN-γ+
ns
Responder
Non-responder
D CD4+ T cells
CD8+ T cells
% GzmB+
ns
Responder
Non-responder
E IFN-γ
Cytokine secretion (pg/ml)
*
ns
- + - + αCD3
Responder
Non-responder
F IFN-γ PBMCs
Cytokine secretion (pg/ml)
-
α-PD1
ns
Control
IFN-α

SI Fig.3 Similar pre-treatment T cell characteristics in responders and non-responders

a) Ratio of alive $CD4^+/CD8^+$ within the $CD3^+$ T cells in treatment-naïve PBMCs of clinical responders (n=12) and non-responders (n=10).

b) PD-1 expression as percentage of alive $CD3^+$T cells, $CD3^+CD4^+$ or $CD3^+CD8^+$ T cells in treatment-naïve PBMCs of clinical responders (n=12) and non-responders (n=10).

c-d) 4 hour PMA/ionomycin stimulated PBMCs of clinical responders (n=12) and non-responders (n=10). Percentage IFN-γ^+ (**c**) or Granzyme B (**d**) expression in alive $CD3^+$ $CD4^+$, and $CD8^+$ T cells.

a-d) Data plotted as boxplots with medians and IQR. Significance determined with Mann-Whitney tests.

e) IFN-γ secretion measured by ELISA at day 6 after start of a MLR with treatment-naïve PBMCs of clinical responders (n=6) and non-responders (n=5), untreated or αCD3 treated (0.1 µg/ml). Experiments were performed in triplo per "n", and medians were calculated. Medians are plotted as boxplots with medians and IQR. Significance determined with T tests.

f) IFN-γ secretion measured by ELISA at day 6 after start of a MLR with PBMCs of control donors, untreated or PD-1 blockade treated (αPD1; 10 µg/ml). The MLR was exposed to medium (control), or 100 U/ml IFN-α (n=3). Experiments were performed in triplo per "n", and medians were calculated. Medians are plotted as floating bar with mean. Significance determined with Wilcoxon tests.

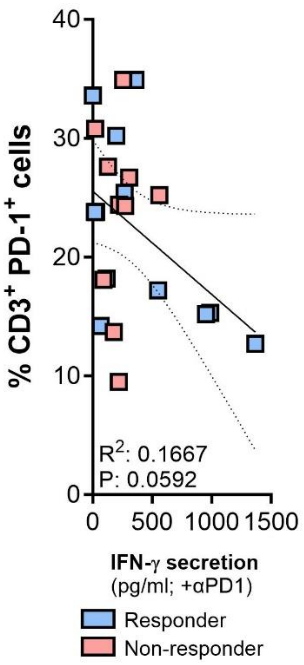

SI Fig.4 PD-1 expression on T cells does not correlate with αPD-1 induced IFN-γ secretion in an MLR

Correlation of percentage $CD3^+PD\text{-}1^+$T cells "SI Fig.3b" with IFN-γ secretion induced by PD-1 blockade "Fig.3b" of treatment-naïve PBMCs of clinical responders (n=12) and non-responders (n=10). Significance determined with simple linear regression.
